# Viral Triggered Celiac Disease: A Case Report

**DOI:** 10.7759/cureus.40429

**Published:** 2023-06-14

**Authors:** Nicole L Welch, Tyler Welch, Busara Songtanin

**Affiliations:** 1 Internal Medicine, Texas Tech University Health Sciences Center Lubbock Campus, Lubbock, USA; 2 Family Medicine, Baptist Health-UAMS (University of Arkansas for Medical Sciences), North Little Rock, USA

**Keywords:** epstein barr virus, cytomegalovirus (cmv), oral tolerance, autoimmune, ebv, mononucleosis, celiac disease

## Abstract

Celiac disease (CD) is an autoimmune condition presenting with a wide variety of nonspecific gastrointestinal symptoms. It can be difficult to diagnose due to the vagueness of complaints such as diarrhea, anemia due to malabsorption, vitamin or electrolyte deficiencies, and/or failure to thrive. This condition is characterized by a sensitivity to ingested gluten-containing compounds. Blood tests can be used for screening, though confirmatory testing by a small intestine biopsy is needed for diagnosis.

Viral infections can trigger autoimmune conditions in individuals. It is possible that viral infections, such as Ebsetein-Barr virus(EBV) or Cytomegalovirus (CMV), can trigger the clinical presentation of celiac disease in certain individuals with genetic predispositions. Early recognition of celiac disease is important to prevent both short and long-term complications and improve the quality of life for the individual. Here, we discuss a case where the patient developed celiac disease only months after a diagnosis of mononucleosis.

## Introduction

Celiac disease (CD), also known as gluten-sensitive enteropathy, is an immune-mediated disease that typically presents with gastrointestinal symptoms, including diarrhea, abdominal pain, and abdominal distension [1,2]. Though once considered a rare disease, increased awareness and screening have resulted in more recent studies showing a prevalence of 0.71-1% in the US [1,2]. This disease can present at any age, though classically within the first few years of life [2]. Diagnosis may be delayed due to nonspecific symptoms or a mild or asymptomatic presentation [2]. While blood screenings are available, confirmatory diagnosis requires a small intestine biopsy with histologic evidence of CD. Dietary avoidance of gluten-containing foods is the only treatment currently available [2].

Certain individuals with CD do not develop signs or symptoms until much later in life. Speculation about the trigger for CD has been an ongoing topic of research. Viral infections, such as Epstein-Barr virus (EBV) and Cytomegalovirus (CMV), have been implicated in triggering autoimmune conditions such as systemic lupus erythematosus [3,4]. It has also been demonstrated that other viral infections have been associated with removing the immune tolerance to orally ingested antigens in CD, which has been referred to as breaking oral tolerance [5,6]. With this knowledge, it can be postulated that certain viral infections may trigger CD in a genetically predisposed population.

## Case presentation

A healthy 19-year-old female with a past medical history of allergic rhinitis presented to the clinic with persistent oral ulcers for approximately two weeks. She complained of pain while chewing salty and acidic foods. The patient reported the ulcers appearing acutely approximately two weeks before presentation, with no prior history of ulcers. The patient had recently been clinically diagnosed with mononucleosis (fevers, chills, pharyngitis, and severe fatigue lasting >2 weeks) two months prior and had made a full recovery with the exception of some lingering fatigue.

Vital signs were unremarkable, the physical exam was notable for circular, shallow-based ulcers on the gingival lining. Laboratory workup shown in Table 1 revealed a hemoglobin level of 118 g/L (normal 120-160 g/L), vitamin D level of 93 nmol/L (normal 75-250 nmol/L) with erythrocyte sedimentation rate (ESR) 6 mm/hr (normal < 20 mm/hr), C-reactive protein (CRP) 1.1 mg/L (normal <8 mg/L), ferritin 34 microg/L (normal 80-300 microg/L), and vitamin B12 237 pmol/L (normal >220 pmol/L).

**Table 1:** Laboratory study results with normal ranges The patient's laboratory values at the time of initial evaluation. The results showed an elevated tissue transglutaminase antibody IgA and endomysial antibody IgA. ESR - erythrocyte sedimentation rate. CRP - C-reactive protein

Test	Result		Normal range
Hemoglobin	118 g/L		120-160 g/L
Vitamin D	93 nmol/L		75-250 nmol/L
Vitamin B12	237 pmol/L		>220 pmol/L
ESR	6 mm/hr		< 20 mm/hr
CRP	1.1 mg/L		<8 mg/L
Ferritin	34 microg/L		80-300 microg/L
Antinuclear antibody	1:80 with a speckled pattern		
Immunoglobulin A	1.84 g/L		0.7-3.8 g/L
Transglutaminase antibody IgA	32 U/mL		0-20U/mL
Endomysial antibody IgA	1:32		< 1:5

Further investigation revealed that the patient had been eating an increased amount of bread products in the past two months. She denied any abdominal pain, diarrhea, nausea, vomiting, heartburn, dysphagia, or fatigue. She also had a family history significant for Hashimoto’s thyroiditis, type 1 diabetes mellitus, rheumatoid arthritis, irritable bowel disease, and psoriasis.

Additional laboratory testing, shown in Table 1, revealed an antinuclear antibody of 1:80 with a speckled pattern, immunoglobulin A (IgA) 1.84 g/L (normal 0.7-3.8 g/L), transglutaminase antibody IgA (TTG IgA) of 32 U/mL (normal 0-20U/mL), and an endomysial antibody IgA (EMA) 1:32 (normal < 1:5 [7]). Esophagogastroduodenoscopy (EGD) appeared to show a normal esophagus, stomach, and duodenum, however, duodenal biopsy results confirmed a diagnosis of CD.

The patient was referred to a dietitian for diet management and started on a gluten-free diet, calcium, and vitamin D supplements. Her oral ulcers were reported to have resolved by her six-month follow-up, and the patient reported strict compliance with her new diet. She also reported new symptoms of nausea, vomiting, and abdominal discomfort after accidental ingestion of gluten. After one year of a gluten-free diet, the patient received a dual-energy X-ray absorptiometry test (DEXA) to assess her bone status, which was normal.

## Discussion

CD, an immune-mediated disorder triggered by gluten-containing foods (those containing wheat, rye, or barley), is a relatively common condition occurring in approximately 1% of the US population [1,2]. Patients can have a wide range of non-specific symptoms, including diarrhea, abdominal pain, and bloating. Other manifestations can include failure to thrive, anemia, vitamin deficiencies, aphthous stomatitis, dermatitis herpetiformis, and psychiatric conditions (depression and anxiety) [2,8].

CD must have both an environmental trigger (gluten peptide-containing foods) and a genetic predisposition [2,7]. It is noteworthy that specific genetic conditions like type 1 diabetes mellitus, autoimmune thyroid disease, Turner syndrome, and Down syndrome, as well as having a first-degree family member with CD, have demonstrated a higher occurrence of CD [2]. This increased prevalence is estimated to be approximately 5-10% [2]. Approximately 98% of patients have either the HLA-DQ2 (90%) or HLA-DQ8 (8-10%) gene loci present on evaluation [2]. The combination of HLA prevalence and autoimmune associations with CD makes it likely there is a significant genetic component to the disease.

CD is a T-cell (TC) mediated disease where T-cells (TC) and dendritic cells (DC) have been sensitized to the gliadin protein found in gluten [5,6]. When gluten is ingested, major histocompatibility class II (MHC II) molecules present the gluten peptide to TC and DC. Through this process, gluten-specific CD4+ cells release anti-gluten and autoimmune antibodies, which subsequently stimulate the release of cytotoxins [5,6,9]. These cytotoxins target the intestinal epithelium leading to epithelial cell death and the characteristic blunted villi seen on histological examination [5]. Blunted villi inhibit effective nutrient absorption leading to nutritional deficiencies.

Oral tolerance is the process of immune nonresponsiveness to ingested foods [5]. Typically, when food is ingested, it is broken down by the gastrointestinal tract and absorbed by the microvilli in the small intestine. The peptides are then taken up by the DC, which stimulates T regulatory (T_reg_) cells [5]. The T_reg_ cells then have an inhibitory response on the inflammation cascade for the specific ingested food [5]. This process is abnormal in patients with CD. Instead of the DC stimulating T_reg_ cells, the DC stimulates gluten-specific CD4+ cells as previously discussed. It has been hypothesized that viral infections stimulate natural killer (NK) pathways, leading to the activation of type 1 interferons (IFNs) resulting in the breaking of oral tolerance [5,6]. Through the breaking of oral tolerance, an immune response to food is initiated leading to cytotoxin production and food-specific antibodies when that food is ingested.

Recent research has shown a relationship between certain viral infections, such as Reovirus, CMV, and EBV, with the subsequent development of autoimmune diseases, including type 1 diabetes mellitus, idiopathic arthritis, and CD, among others, though the precise role remains unclear [4,10-12]. It has been well-documented that EBV can cause epigenetic reprogramming of proteins via methylation of DNA and histone changes, both of which can change the way some genes are expressed [13]. CMV, on the other hand, can cause mutations in toll-like receptors (TLRs), a type of protein that can be found in several innate immune cells, including T-cells [4,12]. These TLRs can mediate inflammation via both the innate and adaptive immune pathways [4,12]. A recent paper by Talipova et al. suggests that a relationship may exist between the gliadin peptide in CD and the activation of pro-inflammatory cascades involving some genetic variants of TLRs [12].

Given this knowledge, it is possible those with genetic predispositions (such as HLA-DQ2 or DQ8) or those with strong personal or family histories of other autoimmune conditions can have genetic modifications take place after a viral infection that triggers the body to become intolerant to the gluten protein and develop CD. Despite the association depicted by this case, more research is needed in this area to further examine possible links between viral infections and the development of CD.

## Conclusions

While acknowledging certain limitations, such as the absence of serology or monospot testing in the clinical diagnosis of mononucleosis, the main objective of this case report is to highlight a potential association or link between viral infections and CD. Individuals with a family history of autoimmune diseases may have a genetic predilection to developing CD. Though this case in no way proves an association, it illustrates the correlation between the development of CD and certain viral infections. More studies investigating the correlation between viral infections, HLA predispositions, TLR, and diagnosis of CD are needed to confirm this hypothesis. Further retrospective case studies should be considered in the future. However, keeping this in mind will allow physicians to have a lower threshold for screening in this cohort of patients. Earlier detection of CD allows a gluten-free diet to be implemented sooner, decreasing the risk and severity of complications.

## References

[1] Rubio-Tapia A, Ludvigsson JF, Brantner TL, Murray JA, Everhart JE (2012). The prevalence of celiac disease in the United States. Am J Gastroenterol.

[2] Barker JM, Liu E (2008). Celiac disease: pathophysiology, clinical manifestations, and associated autoimmune conditions. Adv Pediatr.

[3] Jog NR, James JA (2020). Epstein Barr virus and autoimmune responses in systemic lupus erythematosus. Front Immunol.

[4] Janahi EM, Das S, Bhattacharya SN (2018). Cytomegalovirus aggravates the autoimmune phenomenon in systemic autoimmune diseases. Microb Pathog.

[5] Brown JJ, Jabri B, Dermody TS (2018). A viral trigger for celiac disease. PLoS Pathog.

[6] Abadie V, Sollid LM, Barreiro LB, Jabri B (2011). Integration of genetic and immunological insights into a model of celiac disease pathogenesis. Annu Rev Immunol.

[7] Schuppan D (2000). Current concepts of celiac disease pathogenesis. Gastroenterology.

[8] Kurppa K, Räsänen T, Collin P (2012). Endomysial antibodies predict celiac disease irrespective of the titers or clinical presentation. World J Gastroenterol.

[9] López Casado MÁ, Lorite P, Ponce de León C, Palomeque T, Torres MI (2018). Celiac disease autoimmunity. Arch Immunol Ther Exp (Warsz).

[10] Harley JB, Chen X, Pujato M (2018). Transcription factors operate across disease loci, with EBNA2 implicated in autoimmunity. Nat Genet.

[11] Houen G, Trier NH (2020). Epstein-Barr virus and systemic autoimmune diseases. Front Immunol.

[12] Talipova D, Smagulova A, Poddighe D (2022). Toll-like receptors and celiac disease. Int J Mol Sci.

[13] Scott RS (2017). Epstein-Barr virus: a master epigenetic manipulator. Curr Opin Virol.

